# *Opuntia dillenii* (Ker Gawl.) Haw., Seeds Oil Antidiabetic Potential Using In Vivo, In Vitro, In Situ, and Ex Vivo Approaches to Reveal Its Underlying Mechanism of Action

**DOI:** 10.3390/molecules26061677

**Published:** 2021-03-17

**Authors:** Mohamed Bouhrim, Hayat Ouassou, Salima Boutahiri, Nour Elhouda Daoudi, Hamza Mechchate, Bernard Gressier, Bruno Eto, Hamada Imtara, Amal A. Alotaibi, Mohammed Al-zharani, Abderrahim Ziyyat, Hassane Mekhfi, Abdelkhaleq Legssyer, Mohammed Aziz, Mohamed Bnouham

**Affiliations:** 1 Laboratory of Bioresources, Biotechnology, Ethnopharmacology, and Health, Faculty of Sciences, Mohammed First University, Oujda B.P. 717, Morocco; mohamed.bouhrim@gmail.com (M.B.); hayatouassou@gmail.com (H.O.); Nourelhoudada95@gmail.com (N.E.D.); ziyyat@yahoo.fr (A.Z.); hmekhfi@yahoo.fr (H.M.); alegssyer@yahoo.fr (A.L.); azizmo5@yahoo.fr (M.A.); 2 Research Team on the Chemistry of Bioactive Molecules and Environment, Faculty of Sciences, Moulay Ismaïl University, Meknes, B.P. 11201 Zitoune Meknes, Morocco; boutahirisalima@gmail.com; 3 Laboratory of Biotechnology, Environment, Agrifood, and Health, University of Sidi Mohamed Ben Abdellah, Faculty of Sciences Dhar el Mahraz, Fez B.P. 1796, Morocco; 4 Laboratory of Pharmacology, Pharmacokinetics, and Clinical Pharmacy, Faculty of Pharmaceutical and Biological Sciences, B.P. 83 Lille, France; bernard.gressier@univ-lille2.fr (B.G.); Etobrunoalves1@hotmail.fr (B.E.); 5 Laboratories-TBC, Faculty of Pharmaceutical and Biological Sciences, B.P. 83 Lille, France; 6 Faculty of Arts and Sciences, Arab American University Palestine, Jenin 240, Palestine; hamada.tarayrah@gmail.com; 7 Basic Science Department, College of Medicine, Princess Nourah bint Abdulrahman University, Riyadh 11671, Saudi Arabia; amaalotaibi@pnu.edu.sa; 8 Biology Department, College of Science, Imam Mohammad ibn Saud Islamic University (IMSIU), Riyadh 11623, Saudi Arabia; mmyalzahrani@imamu.edu.sa

**Keywords:** *Opuntia dillenii*, seeds oil, diabetes mellitus, antihyperglycemic, streptozotocin, intestinal glucose absorption, Ussing chamber, intestinal α-glucosidase, pancreatic α-amylase, biological activity, medicinal plant

## Abstract

*Opuntia dillenii* Ker Gawl. is one of the medicinal plants used for the prevention and treatment of diabetes mellitus (DM) in Morocco. This study aims to investigate the antihyperglycemic effect of *Opuntia dillenii* seed oil (ODSO), its mechanism of action, and any hypoglycemic risk and toxic effects. The antihyperglycemic effect was assessed using the OGTT test in normal and streptozotocin (STZ)-diabetic rats. The mechanisms of action were explored by studying the effect of ODSO on the intestinal absorption of d-glucose using the intestinal in situ single-pass perfusion technique. An Ussing chamber was used to explore the effects of ODSO on intestinal sodium-glucose cotransporter 1 (SGLT1). Additionally, ODSO’s effect on carbohydrate degrading enzymes, pancreatic α-amylase, and intestinal α-glucosidase was evaluated in vitro and in vivo using STZ-diabetic rats. The acute toxicity test on mice was performed, along with a single-dose hypoglycemic effect test. The results showed that ODSO significantly attenuated the postprandial hyperglycemia in normal and STZ-diabetic rats. Indeed, ODSO significantly decreased the intestinal d-glucose absorption in situ. The ex vivo test (Ussing chamber) showed that the ODSO significantly blocks the SGLT1 (IC_50_ = 60.24 µg/mL). Moreover, ODSO indu\ced a significant inhibition of intestinal α-glucosidase (IC_50_ = 278 ± 0.01 µg/mL) and pancreatic α-amylase (IC_50_ = 0.81 ± 0.09 mg/mL) in vitro. A significant decrease of postprandial hyperglycemia was observed in sucrose/starch-loaded normal and STZ-diabetic ODSO-treated rats. On the other hand, ODSO had no risk of hypoglycemia on the basal glucose levels in normal rats. Therefore, no toxic effect was observed in ODSO-treated mice up to 7 mL/kg. The results of this study suggest that ODSO could be suitable as an antidiabetic functional food.

## 1. Introduction

Diabetes mellitus is chronic metabolic trouble marked by a high blood sugar level due to abnormal insulin synthesis or action [1,2]. Among the most well-known types of diabetes is type 1 diabetes, where the body does not produce insulin or in insufficient quantities, which develops due to lifestyle changes, viral infections, toxins ingestion, and genetic predisposition. Moreover, type 2 diabetes is due to insulin resistance leading to long-term insulin deficiency. It is strongly related to obesity or overweight, increasing age, ethnicity, and family history [3]. Currently, the global prevalence of diabetes is 9.3%; this number will reach 10.2% by 2030 and 10.9% by 2045 [4]. Globally, about 50% of diabetes cases are undiagnosed owing to the unawareness of symptoms, and the majority of these cases come from low- and middle-income countries [5]. Furthermore, poor blood d-glucose control in diabetic patients leads to permanent hyperglycemia [6]. In diabetic patients, postprandial hyperglycemia is involved in plasmatic and cellular proteins glycation, which take part in the development of diabetes complications [7,8,9]. In this regard, diabetes mellitus (DM) management requires accurate postprandial glycemic control through decreasing the glucose absorption [10]. This is possible via the inhibition of carbohydrate enzymes and/or inhibition of glucose absorption by the intestinal epithelial cells [9,10]. Different treatments are currently used to manage diabetes, such as insulin, dietetic therapies, and pharmacotherapy, which exert antidiabetic effects by different mechanisms. These mechanisms include increasing d-glucose’s cellular intake by biguanides and thiazolidinediones, stimulating insulin secretion by sulfonylureas and meglitinides, delaying carbohydrates absorption from the luminal intestinal space by intestinal α-glucosidase inhibitors, and reducing hepatic gluconeogenesis by biguanides [11,12,13]. Since antiquity, people have used plants not only to perfume themselves, clothe themselves, feed themselves, etc. but, also, to heal themselves [14]. Indeed, thanks to the availability, low cost, and therapeutic effectiveness of these natural products, the population rate that uses medicinal plants to treat disease spills is high and estimated as 80% according to a WHO report [15]. Many medicinal plants are used traditionally in the management of diabetes throughout the world [16,17]. Marles and Farnsworth estimated that more than 1000 plant species are being used against DM [18]. The efficacy and safety of some plants have been sufficiently validated by clinical use over thousands of years [19]. The *Opuntia* plant belongs to the family of Cactaceae [20]. It originated from Mexico and was first observed in North Africa during the sixteenth century [21]. Actually, up to 1500 species belong to the *Opuntia* genus, and they are distributed in several regions in the world, like the Mediterranean countries, Europe, and sub-Saharan Africa [22]. The majority of these species are characterized by the production of edible and aromatic fruits [23]. This *Opuntia* plant spreads wildly in arid and semi-arid regions, where there is a high use and low demand for water to increase the production of *Cactus*, as highlighted by the Food and Agriculture Organization [24]. *Opuntia dillenii* Ker Gawl. is a *Cactus* species, growing in Western and Northeastern Morocco. It plays an important role in subsistence agriculture. The *Opuntia dillenii* fruit is characterized by a sour taste and the presence of a large number of seeds. This plant is used in alternative medicine to treat many diseases, such as DM [25,26,27]. *Opuntia dillenii* is used in traditional medicine to treat diabetes [28]. In Tunisia, the ripe fruit of *opuntia dillenii* is used in traditional medicine as an antidiabetic [29]. In Pakistan, *Opuntia dillenii* is also used for the treatment of diabetes [30]. In traditional Canadian medicine, the fruit of *Opuntia dillenii* is used as an antidiabetic agent [31]. However, little interest has been given to the use of the fruit seeds by traditional medicine, because the body is unable to digest them and, therefore, to benefit from their constitutions. Several scientific studies have been interested in studying the different vegetative parts of *Opuntia* to assess or confirm their beneficial biological effects on various diseases. Over the last few years, researchers have been interested in studying the seed oil of *Opuntia dillenii* and have found that they are rich in very active molecules [26,32].

*Opuntia dillenii* seed oil contains a high amount of linoleic acid, β-sitosterol, and γ-tocopherols, the main components of constitutive fatty acids, phytosterols, and tocopherols, respectively [33]. These phytochemical compounds have shown very important antidiabetic properties in several studies [34,35]. Previous papers have reported the potential bioeffects of ODSO, including antioxidant, anti-inflammatory, and hepatoprotective activities [36,37]. Furthermore, in a previous work, the antidiabetic effect of ODSO was studied, but the mechanism of action still has not been elucidated [38,39]. The present work aims to study the postprandial glycemic control by ODSO through an oral glycemic load shedding light on the possible mechanisms during the absorptive process by targeting digestive enzymes (α-amylase and α-glucosidase) and glucose movement across the intestinal epithelial barrier.

## 2. Results

### 2.1. Yield of Extraction

The maceration of *Opuntia dillenii* seeds for 24 h with the use of petroleum ether as a lipid extractor gave a yield of 7% (oil component from the dry weight).

### 2.2. Acute Toxicity

The results of this acute toxicity test showed that ODSO is not toxic at a dose up to 7 mL/kg. It did not cause any signs of toxicity (diarrhea, vomiting, abnormal mobility, etc.) or mortality during the entire surveillance period (14 days).

### 2.3. Hypoglycemic Test in Non-Diabetic Rats

**Figure 1:**
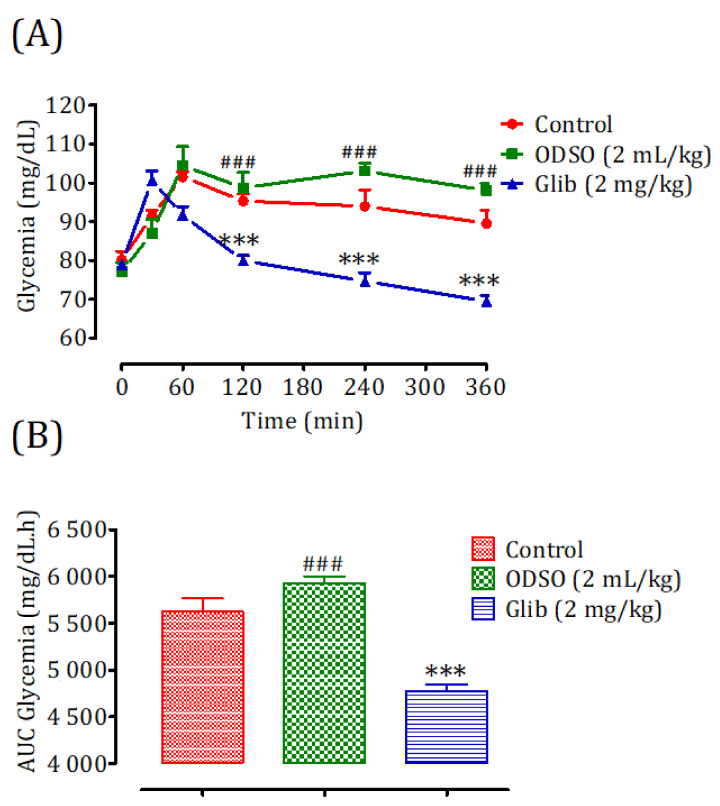
Changes in the basal blood d-glucose (**A**) and area under the basal blood d-glucose curves (**B**) in normal rats after administration of the tested products (ODSO and glibenclamide). ODSO: *Opuntia dillenii* seed oil. The values are the means ± SEM (*n* = 6). ODSO: *Opuntia dillenii* seed oil. *** *p* < 0.01 in comparison with the control and ^###^ *p* < 0.001 compared to the glib group. Glib: glibenclamide and AUC: area under the curve.

The results of this test showed that the administration of ODSO at a dose of 2 mL/kg did not produce a risk of hypoglycemia on the basal blood d-glucose level of nondiabetic rats. The change in blood sugar during the six h after ODSO administration was not significant compared to the control group. In contrast, the glibenclamide caused a significant decrease (*p* < 0.001) during the two, four, and six h, compared to the control group. Moreover, the comparison of the effect of ODSO with that of glibenclamide showed that there has a significant (*p* < 0.001) difference between them at the 120 min, 240, and 360 min (Figure 1A). Besides, the area under the curve (AUC d-glucose) for the ODSO group (5932 ± 169 mg/dL/h) was not significantly different, compared to the control group (5625 ± 169 mg/dL/h). The area under the curve for the glibenclamide group was significantly lower (*p* < 0.001) (4777 ± 184 mg/dL/h) compared to the area under the curve of the control group (5625 ± 169 mg/dL/h). Moreover, there was a significant (*p* < 0.001) difference between the area under the curves of the ODSO and glib groups (Figure 1B).

### 2.4. Antihyperglycemic Study in Nondiabetic and Diabetic Rats

#### 2.4.1. Nondiabetic Rats

**Figure 2:**
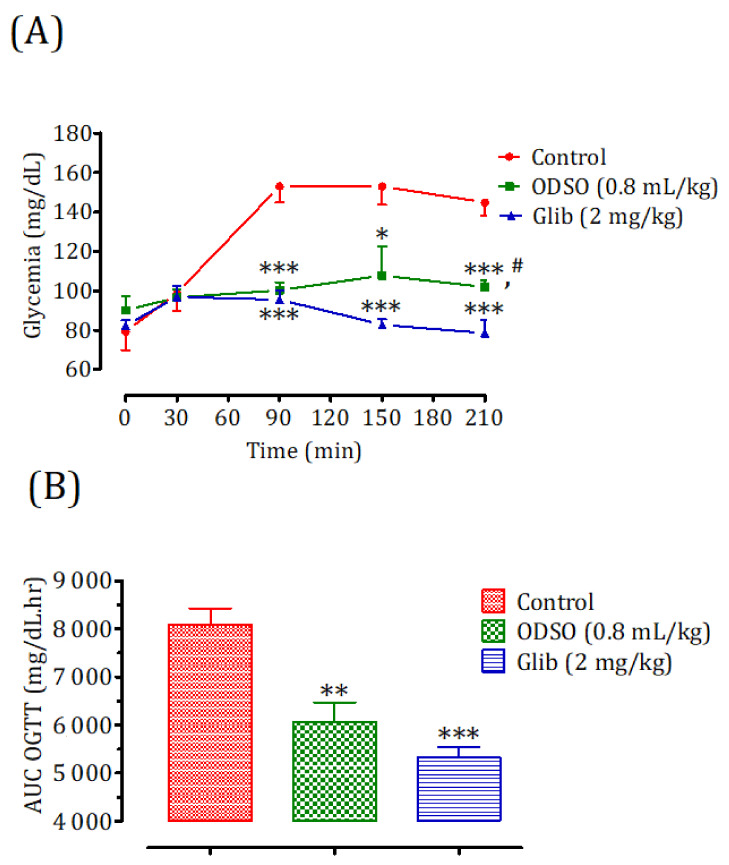
Effect of ODSO and glibenclamide on the change in postprandial glycemia in normal rats (**A**) and with a representation in the area under curves (**B**). ODSO: *Opuntia dillenii* seed oil. The values are the means ± SEM (*n* = 6). * *p* < 0.05, ** *p* < 0.01, and *** *p* < 0.001 compared to the control. ^#^ *p* < 0.05 compared to the glib group.

Oral administration of ODSO at 0.8 mL/kg 30 min prior to d-glucose overload in nondiabetic rats significantly reduced the postprandial hyperglycemia at 90 min (*p* < 0.001; 1 ± 4 g/L), 150 min (*p* < 0.05; 108 ± 15 mg/dL), and 210 min (*p* < 0.001; 103 ± 4 mg/dL) compared with the group of rats pretreated with distilled water in which a d-glucose overload-induced remarkable hyperglycemia was observed: at 90 min (153 ± 8 mg/dL), 150 min (153 ± 9 mg/dL), and 210 min (145 ± 7 mg/dL). Similarly, glibenclamide significantly inhibited postprandial hyperglycemia for three h following the d-glucose overload at 90 min (*p* < 0.001; 95 ± 4 mg/dL), 150 min (*p* < 0.001; 83 ± 3 mg/dL), and 210 min (*p* < 0.001; 78 ± 6 mg/dL) compared with the group of rats pretreated with distilled water. Moreover, the comparison of the effect of ODSO with that of glibenclamide showed that it did not have a significant difference between them at 90 min and 150 min, but it was significant after 210 min (*p* < 0.05) (Figure 2A). The area under the curve (AUC d-glucose) was significantly (*p* < 0.01) lower in rats treated with ODSO (6062 ± 1003 mg/dL/h) than in rats treated with distilled water (8098 ± 806 mg/dL/h). Besides, the area under the glibenclamide curve was significantly (*p* < 0.001) lower (5322 ± 545 mg/dL/h) compared to the area under the curve of the distilled water-treated rats (8098 ± 806 mg/dL/h). Moreover, there was not a significant difference between the area under the curves of the ODSO and glib groups (Figure 2B).

#### 2.4.2. Diabetic Rats

**Figure 3:**
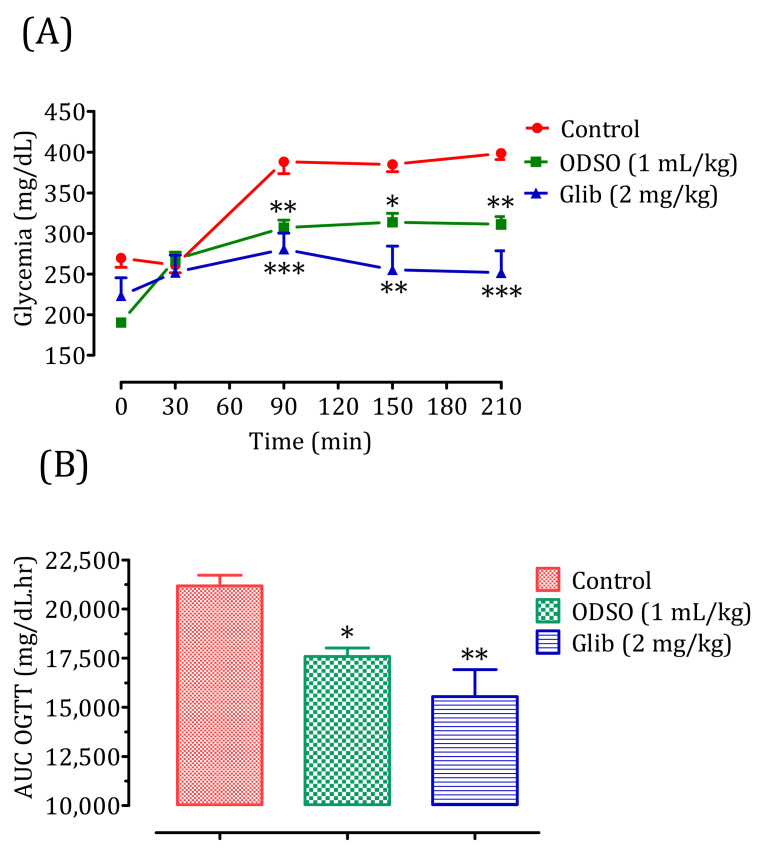
Effect of the oil (ODSO) and glibenclamide (Glib) on the postprandial glycemia variations in diabetic rats (**A**), with a representation of the area under curves (**B**). ODSO: *Opuntia dillenii* seed oil. The values are the means ± SEM (*n* = 6). * *p* < 0.05, ** *p* < 0.01, and *** *p* < 0.001 compared to the control.

The oral administration of ODSO at 1 mL/kg 30 min before the d-glucose overload in diabetic rats significantly reduced the postprandial hyperglycemia at 90 min (*p* < 0.01; 307 ± 8 mg/dL), 150 min (*p* < 0.05; 314 ± 1 mg/dL), and 210 min (*p* < 0.001; 311 ± 9 mg/dL) compared with the group of rats pretreated with distilled water in which a d-glucose overload-induced remarkable hyperglycemia was observed: at 90 min (388 ± 13 mg/dL), 150 min (385 ± 8 mg/dL), and 210 min (398 ± 7 mg/dL). Similarly, glibenclamide significantly inhibited postprandial hyperglycemia during the three h following the d-glucose overload at 90 min (*p* < 0.001; 280 ± 18 mg/dL), 150 min (*p* < 0.01; 255 ± 26 mg/dL), and 210 min (*p* < 0.001; 251 ± 25 mg/dL) compared with the group of rats pretreated with distilled water. Moreover, the comparison of the effect of ODSO with that of glibenclamide showed that there was not a significant difference between them at 90 min, 150 min, and 210 min (Figure 3A). The area under the curve (AUC d-glucose) was significantly (*p* < 0.05) lower in rats pretreated with ODSO (17,576 ± 987 mg/dL/h) than in rats pretreated with distilled water (21,181 ± 1204 mg/dL/h). Additionally, the glibenclamide area under the curve was significantly (*p* < 0.01) lower (15,545 ± 3079 mg/dL/h) compared to the area under the curve of rats pretreated with distilled water (21,181 ± 1204 mg/dL/h). Moreover, there was not a significant difference between the area under the curves of the ODSO and glib groups (Figure 3A).

### 2.5. Intestinal Absorption Inhibition of d-Glucose, In Situ

**Figure 4:**
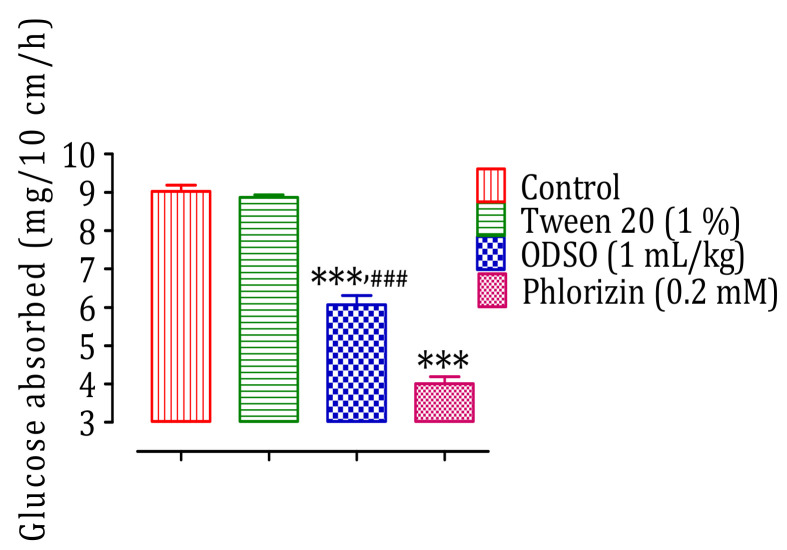
Inhibitory effect of the intestinal absorption of d-glucose by oil (ODSO) and phlorizin in situ. ODSO: *Opuntia dillenii* seed oil. The values are the means ± SEM (*n* = 6). *** *p* < 0.001 compared to the control and ^###^ *p* < 0.001 compared to the Phlorizin group.

The results of this test showed that ODSO at a dose of 1 mL/kg had a significant inhibitory effect of the intestinal absorption of d-glucose in normal Wistar rats (*p* < 0.001; 6.07 ± 0.55 mg/10 cm/h) in situ compared with the control group (9.02 ± 0.36 mg/10 cm/h). Besides, the antidiabetic drug phlorizin also had a significant inhibitory effect on the intestinal absorption of d-glucose (*p* < 0.001; 4.01 ± 0.41 mg/10 cm/h). Moreover, the effect of ODSO was significantly (*p* < 0.001) lower than that of phlorizin (Figure 4).

### 2.6. The ODSO Inhibits the Sodium-Dependent Absorption of d-Glucose

**Figure 5:**
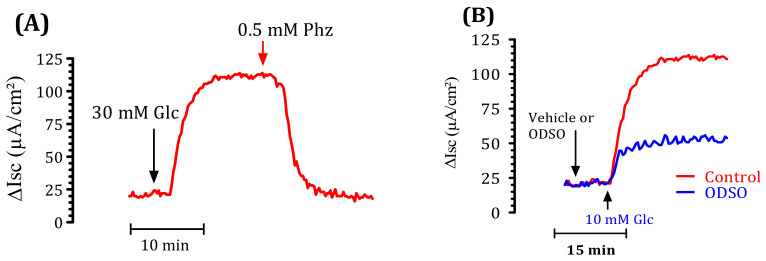

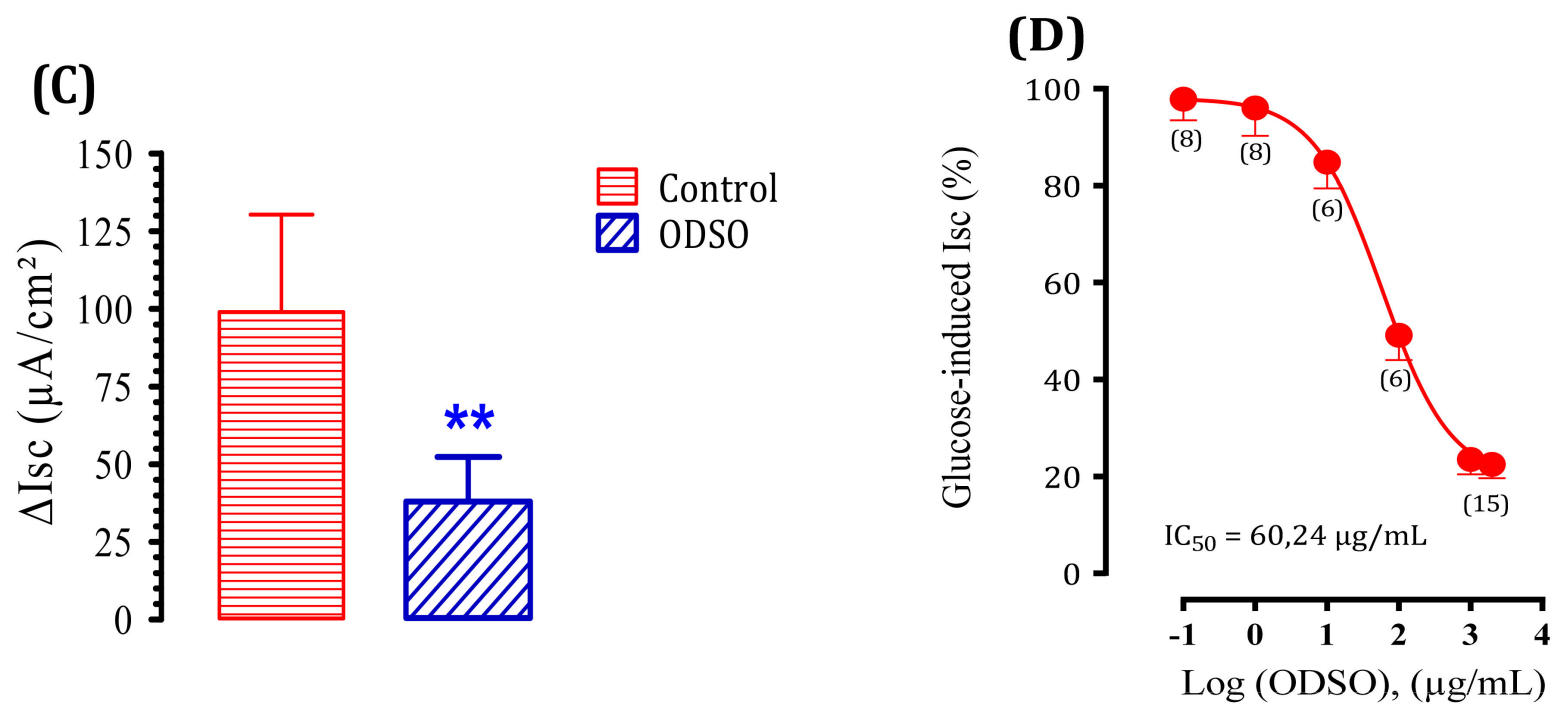
Effect of ODSO on the glucose-induced short-circuit current (Isc). (**A**) Typical recording of the inhibition of Isc (μA/cm^2^) across the mouse jejunum segment isolated in the Ussing chamber by phlorizin (PHZ) added on the mucosal side 10 min before the luminal d-glucose challenge (30 mM). This figure showed that PHZ induced the total inhibition of Isc. (**B**) ODSO (1 mg/mL) diluted in a Ringer solution was added on the mucosal side 10 min before the luminal d-glucose challenge (30 mM). An increase in Isc reflects glucose-related electrogenic sodium absorption through (Na+)-glucose cotransporter-1, SGLT1. The maximal increase in Isc was measured at the plateau. (**C**) The AUC of the vehicle and ODSO effects on d-glucose induced Isc. (**D**) The concentration-response inhibition of glucose-induced Isc is expressed as a % of the maximum effect induced by 0.5 mM of phlorizin (PHZ); *n* = 6–15 ** *p* < 0.001 tissues studied.

We used a short-circuit technique in an Ussing chamber to characterize the effect of ODSO on intestinal glucose transport. The effect of ODSO on the electrogenic intestinal absorption of d-glucose is shown in Figure 5. After reaching a steady state (at least 40 min), mouse jejunal mucosa was challenged on the mucosal side with 30-mM d-glucose (Figure 5A). The results showed that the introduction of ODSO five min before the addition of d-glucose attenuated the increase in the short-circuit current induced by d-glucose (Figure 5B). The area under the curve of the d-glucose absorption after the introduction of ODSO (37.97 ± 14.40 µA/cm^2^) was significantly (*p* < 0.01) lower than the area under the curve of the d-glucose absorption without the addition of ODSO (98.93 ± 31.30 µA/cm^2^) (Figure 5C). The introduction of ODSO into the mucosal reservoir five min before the addition of d-glucose induced a significant dependent concentration inhibition of the short-circuit current (Isc) (IC_50_ = 60.24 µg/mL), as shown in Figure 5D.

### 2.7. Inhibition Assay of Pancreatic α-Amylase Activity In Vitro

**Figure 6:**
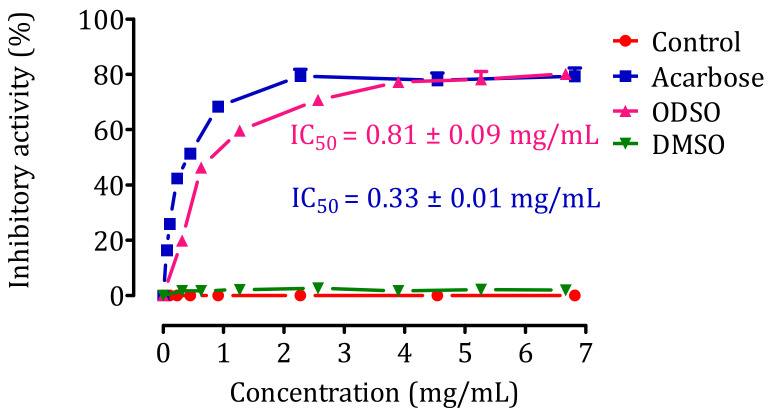
Inhibitory effect of pancreatic α-amylase activity by ODSO and acarbose in vitro. The values are the means ± SEM (*n* = 3). ODSO: *Opuntia dillenii* seed oil.

The result of the effect of ODSO on the activity of pancreatic α-amylase in vitro is shown in Figure 6. Indeed, the activity of the pancreatic α-amylase enzyme was significantly (*p <* 0.001) inhibited in the presence of ODSO. The inhibitory effect of the pancreatic α-amylase enzyme is proportional to the ODSO concentration, with an IC_50_ = 0.81 ± 0.09 mg/mL compared with the control test. Besides, this inhibitory effect of α-amylase by ODSO is significantly (*p <* 0.001) lower than that of acarbose, with an IC_50_ = 0.33 ± 0.01 mg/mL.

### 2.8. Inhibition Assay of Pancreatic α-Amylase Activity in Non-Diabetic and Diabetic Rats

#### 2.8.1. Nondiabetic Rats

**Figure 7:**
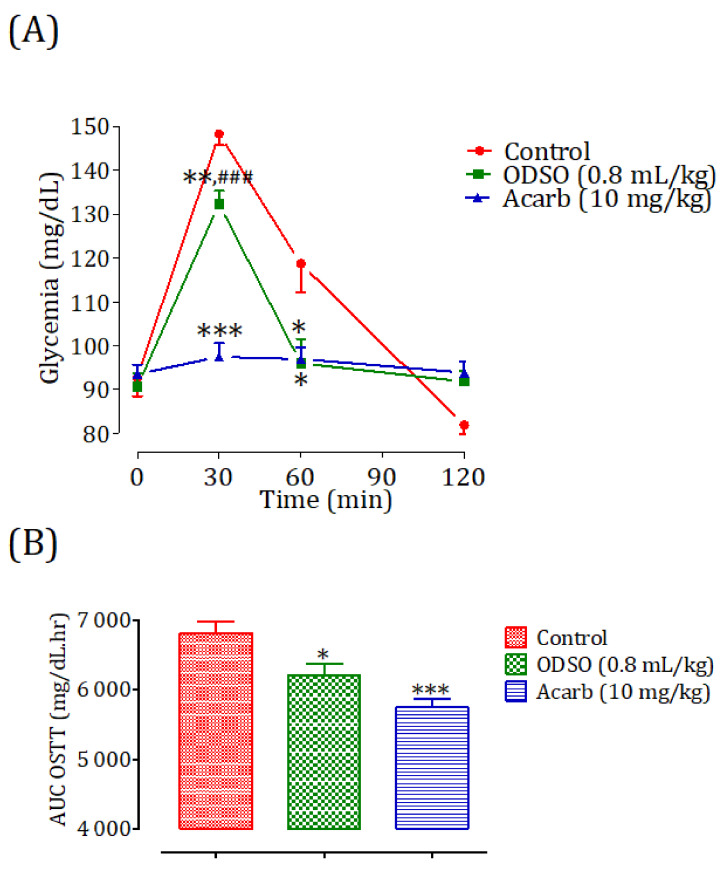
Effect of ODSO and acarbose on the change in the postprandial glycemia in normal rats (**A**), with a representation of the area under the curves (**B**). ODSO: Opuntia dillenii seed oil. The values are the means ± SEM (*n* = 6). * *p* < 0.05, ** *p* < 0.01, and *** *p* < 0.001 compared to the control and ^###^ *p* < 0.00: compared to the acarbose group.

The oral administration of ODSO at 0.8 mL/kg 30 min before a starch overload in nondiabetic rats significantly reduced the postprandial hyperglycemia at 30 min (*p* < 0.01; 132 ± 7 mg/dL) and 60 min (*p* < 0.05; 99 ± 23 mg/dL), and no effect was observed at 120 min in comparison with the group of rats pretreated with distilled water in which a starch overload-induced remarkable hyperglycemia was observed: at 30 min (148 ± 06 mg/dL) and 60 min (118 ± 17 mg/dL). Similarly, the acarbose intake significantly inhibited postprandial hyperglycemia during the two hours following the starch overload at 30 min (*p* < 0.01; 97 ± 6 mg/dL) and 60 min (*p* < 0.01; 97 ± 6 mg/dL) compared to the group of rats pretreated with distilled water. At 120 min, the blood sugar in all groups was similar. Moreover, the comparison of the effect of ODSO with that of acarbose showed that there was a significant difference between them at 30 min and no significant difference at 60 min and 120 min (Figure 7A). The area under the curve (AUC d-glucose) was significantly (*p* < 0.05) lower in rats treated with ODSO (6222 ± 467 mg/dL/h) than in rats treated with distilled water (6822 ± 437 mg/dL/h). Besides, the area under the curve of acarbose was significantly (*p* < 0.001) lower (5754 ± 293 mg/dL/h) compared to the area under the curve of the water-treated rats (6822 ± 437 mg/dL/h). Moreover, there was not a significant difference between the area under the curves of the ODSO and acarbose groups (Figure 7B).

#### 2.8.2. Diabetic Rats

**Figure 8:**
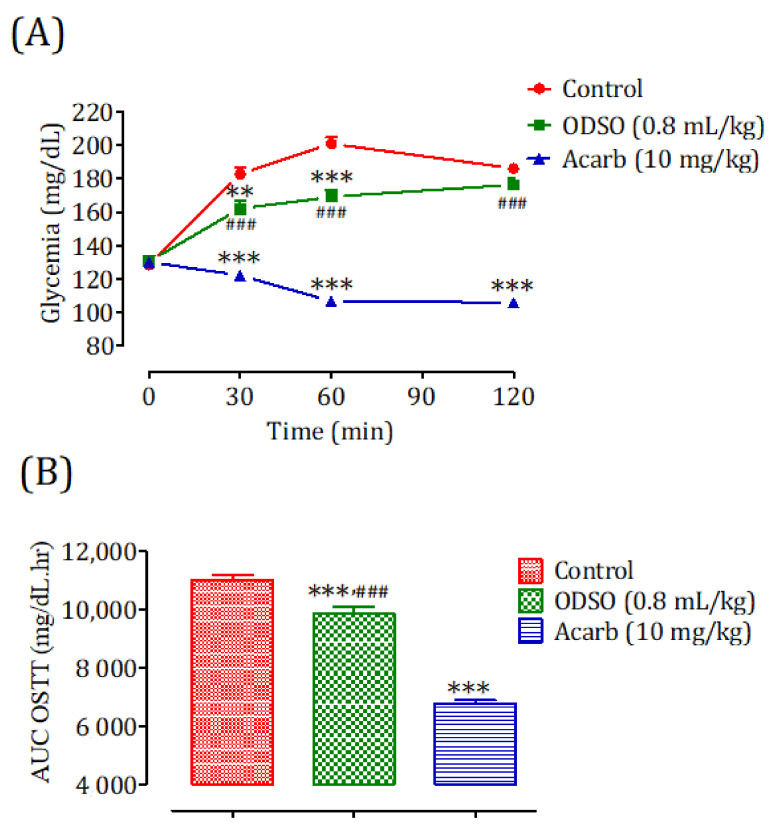
Effect of ODSO and acarbose on the change in the postprandial glycemia in diabetic rats (**A**), with a representation of the area under the curves (**B**). ODSO: *Opuntia dillenii* seed oil. The values are the means ± SEM (*n* = 6). ** *p <* 0.01 and *** *p <* 0.001 compared to the control and ^###^ *p* < 0.001 compared to the acarbose group.

The oral administration of ODSO at 0.8 mL/kg 30 min before the starch overload in diabetic rats significantly reduced the postprandial hyperglycemia to 30 min (*p <* 0.01; 162 ± 12 mg/dL) and 60 min (*p <* 0.001; 169 ± 10 mg/dL). At 120 min, the effect of the oil was not significant (176 ± 10 mg/dL) in comparison with the group of rats pretreated with distilled water in which the starch overload-induced remarkable hyperglycemia was observed: at 30 min (182 ± 10 mg/dL), 60 min (201 ± 10 mg/dL), and 120 min (186 ± 7 mg/dL). Similarly, acarbose significantly inhibited the postprandial hyperglycemia during the two hours following the starch overload at 30 min (*p <* 0.001; 122 ± 7 mg/dL), 60 min (*p <* 0.001; 106 ± 6 mg/dL), and 120 min (*p <* 0.01; 105 ± 4 mg/dL) compared with the group of rats pretreated with distilled water. Moreover, the comparison of the effect of ODSO with that of acarbose showed that there was a significant difference between them at 30 min, 60 min, and 120 min (Figure 8A). The area under the curve (AUC d-glucose) was significantly (*p <* 0.001) lower in rats treated with ODSO (9862 ± 595 mg/dL/h) than that of rats treated with distilled water (11,017 ± 399 mg/dL/h). Additionally, the curve area of acarbose was significantly (*p <* 0.001) low (67.93 ± 2.64 mg/dL/h) compared to the area under the curve of water-treated rats. Moreover, there was a significant (*p <* 0.001) difference between the area under the curves of the ODSO and acarbose groups (Figure 8B).

### 2.9. Inhibition Assay of Intestinal α-Glucosidase Activity In Vitro

**Figure 9:**
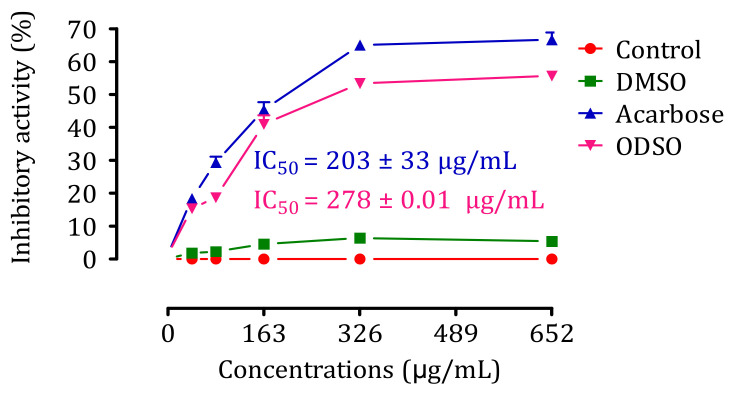
Inhibitory effect of the intestinal α-glucosidase activity by ODSO and acarbose in vitro. The values are the means ± SEM (*n* = 3). ODSO: *Opuntia dillenii* seed oil.

The results of the effect of ODSO on the activity of intestinal α-glucosidase in vitro are shown in Figure 9. Indeed, the activity of the intestinal α-glucosidase enzyme was significantly (*p <* 0.001) inhibited in the presence of ODSO compared to the test control. The inhibitory effect of the intestinal α-glucosidase activity was proportional to the ODSO concentration, with an IC_50_ = 278 ± 0.01 µg/mL compared with the control test. Besides, this inhibitory effect of intestinal α-glucosidase by ODSO was significantly (*p <* 0.001) lower than that of acarbose (IC_50_ = 203 ± 33 mg/mL).

### 2.10. Kinetics of Intestinal α-Glucosidase Inhibition In Vitro

**Figure 10:**
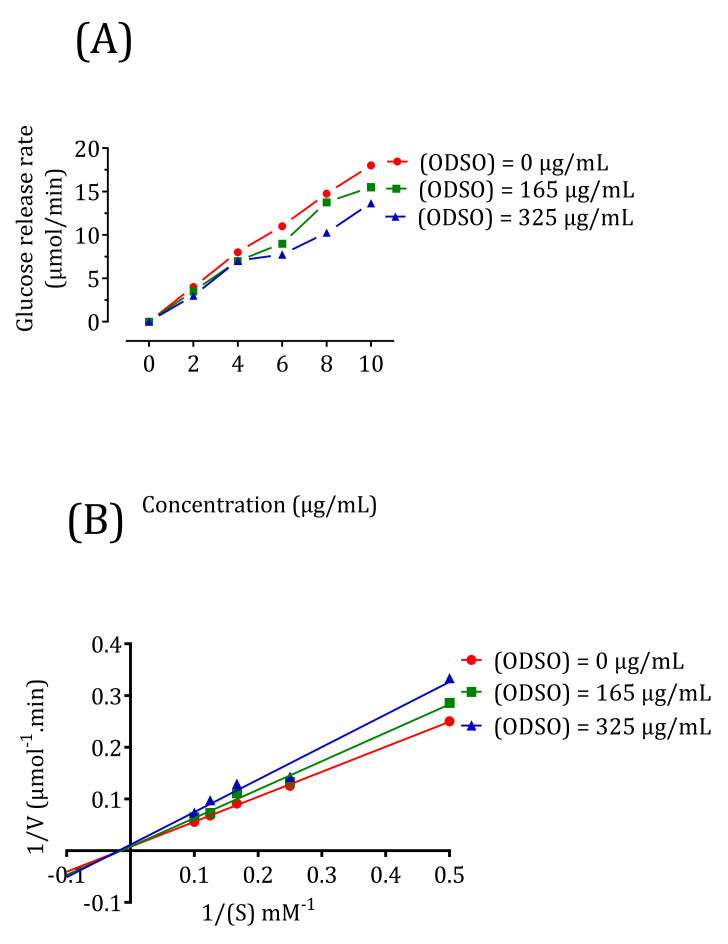
Kinetics of the intestinal α-glucosidase inhibition by ODSO: (**A**) the d-glucose release rate as a function of the sucrose concentration. (**B**) Lineweaver-Burk graph for the kinetic analysis of the intestinal α-glucosidase inhibition by ODSO. ODSO: *Opuntia dillenii* seed oil.

The results in Figure 10 show that the d-glucose release rate decreases in the presence of 165-μg/mL ODSO and continues to decrease after increasing the ODSO dose to 325 μg/mL compared to the control (Figure 10A). To characterize the inhibition process of ODSO against α-glucosidase, the results were analyzed using Lineweaver-Burk waveforms. In Figure 10B, the intersection of the three lines on the horizontal axis showed that ODSO exerts a noncompetitive reversible mode inhibitor effect on the intestinal α-glucosidase enzyme in vitro. The type of noncompetitive reversible inhibition indicates that the change in substrate concentration will not affect the inhibition rate. The Km and Vmax values are shown in Table 1.

### 2.11. Inhibition Assay of Intestinal α-Glucosidase Activity in Nondiabetic and Diabetic Rats

#### 2.11.1. Nondiabetic Rats

**Figure 11:**
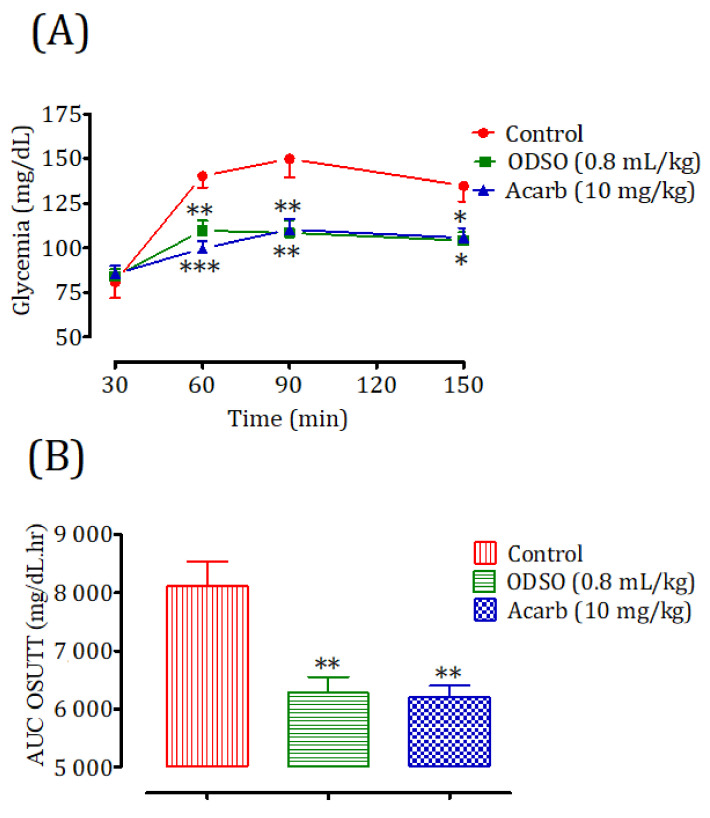
Effect of oil (ODSO) and acarbose (Acarb) on the change in the postprandial glycemia in normal rats (**A**), with a representation of the area under the curves (**B**). The values are the means ± SEM (*n* = 6). * *p* < 0.05, ** *p* < 0.01, and *** *p* < 0.001 compared to the control.

The oral administration of ODSO at 0.8 mL/kg 30 min prior to the sucrose overload in nondiabetic rats significantly reduced the postprandial hyperglycemia at 30 min (*p* < 0.01; 109 ± 4 mg/dL), 60 min (*p* < 0.01; 108 ± 5 mg/dL), and 120 min (*p* < 0.05; 104 ± 4 mg/dL) compared with the group of rats pretreated with distilled water in which the sucrose overload-induced remarkable hyperglycemia was observed: at 30 min (140 ± 6 mg/dL), 60 min (150 ± 10 mg/dL), and 120 min (135 ± 9 mg/dL). Similarly, acarbose significantly inhibited the postprandial hyperglycemia during the two hours following the sucrose overload at 30 min (*p* < 0.001; 99 ± 4 mg/dL), 60 min (*p* < 0.001; 110 ± 6 mg/dL), and 120 min (*p* < 0.001; 106 ± 5 mg/dL) compared with the group of rats pretreated with distilled water. Moreover, the comparison of the effect of ODSO with that of acarbose showed that there was no significant difference between them at 30 min, 60 min, and 120 min (Figure 11A). The area under the curve (AUC d-glucose) was significantly (*p* < 0.01) lower in rats treated with ODSO (6281 ± 661 mg/dL/h) than in rats treated with distilled water (8110 ± 1038 mg/dL/h). Besides, the area under the curve of acarbose was significantly (*p* < 0.01) lower (6205 ± 473 mg/dL/h) compared to the area under the curve of the water-treated rats (8110 ± 1038 mg/dL/h). Moreover, there was no significant difference between the area under the curves of the ODSO and acarbose groups (Figure 11B).

#### 2.11.2. Diabetic Rats

**Figure 12:**
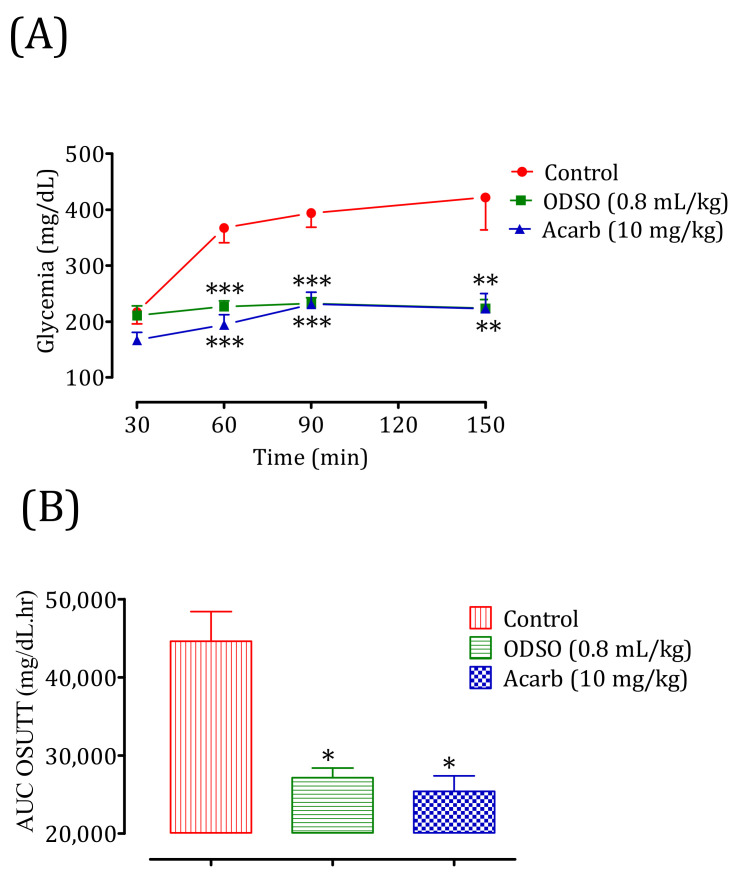
Effect of oil (ODSO) and acarbose (Acarb) on the change in the postprandial glycemia in diabetic rats (**A**), with a representation in the form of the area under the curve (**B**). The values are the means ± SEM (*n* = 6). * *p* < 0.05, ** *p* < 0.01, and *** *p* < 0.001 compared to the control.

The oral administration of ODSO at 0.8 mL/kg 30 min before the sucrose overload in diabetic rats significantly reduced the postprandial hyperglycemia at 30 min (*p* < 0.001; 226 ± 9 mg/dL), 60 min (*p* < 0.001; 232 ± 9 mg/dL), and 120 min (*p* < 0.01; 223 ± 14 mg/dL) compared with the group of rats pretreated with distilled water in which the sucrose overload-induced remarkable hyperglycemia was observed: at 30 min (367 ± 14 mg/dL), 60 min (393 ± 13 mg/dL), and 120 min (421 ± 32 mg/dL). Similarly, acarbose significantly inhibited the postprandial hyperglycemia during the two hours following the sucrose overload at 30 min (*p* < 0.001; 194 ± 16 mg/dL), 60 min (*p* < 0.001; 231 ± 19 mg/dL), and 120 min (*p* < 0.01; 222 ± 24 mg/dL) compared with the group of rats pretreated with distilled water. Moreover, the comparison of the effect of ODSO with that of acarbose showed that there was a significant difference between them at 30 min, 60 min, and 120 min (Figure 12A). The area under the curve (AUC d-glucose) was significantly (*p* < 0.05) lower in rats treated with ODSO (2712 ± 2886 mg/dL/h) than in rats treated with distilled water (44,618 ± 8466 mg/dL/h). Additionally, the area under the curve of acarbose was significantly (*p* < 0.05) low (25,396 ± 4494 mg/dL/h) compared to the area under the curve of water-treated rats (44,618 ± 8466 mg/dL/h). Moreover, there was no significant difference between the area under the curves of the ODSO and acarbose groups (Figure 12B).

## 3. Discussion

Hyperglycemia is the most important feature of DM [40]. The rise of d-glucose in the blood generates oxygen-free radicals through d-glucose auto-oxidation [41] and also induces an increase in the serum levels of advanced glycation products [42]. The lack of control of postprandial hyperglycemia in diabetic patients will lead to the development of severe macro- and microvascular complications [43]. Therefore, controlling this biochemical parameter is a crucial therapeutic step in diabetic patients.

Under normal conditions, postprandial blood d-glucose is linked to two processes: the transformation of long-chain sugars into absorbable units and the intestinal absorption of these carbohydrate units. After a meal, polysaccharides are subjected to the action of α-amylase to transform them into oligosaccharides, and then, the action of intestinal α-glucosidase in the final phase is to give absorbable monosaccharides. To reach the blood, these monosaccharides are transported through the SGLT1 located in the brush border membrane (BBM) to the absorbing epithelial cells. Then, with the aid of the facilitated diffusion (GLUT2) located on the basolateral membrane (BLM) of the epithelial cells, they will cross into the blood [44].

One of the targeted pathways for treating DM is the food digestion route—more specifically, the inhibition of sugar-digesting enzymes such as intestinal α-glucosidase and pancreatic α-amylase and, also, a slowing of the intestinal absorption of d-glucose and a decrease in postprandial d-glucose [45]. The inhibition of digestive enzymes by naturally occurring sources could delay digestion and, thus, decrease d-glucose uptake [46].

In this study, the effect of the oil of *Opuntia dillenii* seeds on postprandial hyperglycemia was studied, and the exploration of its mechanisms of action was realized. The obtained results in our work showed, for the first time, that ODSO could improve d-glucose tolerance by controlling postprandial hyperglycemia caused by d-glucose overload. Indeed, the oral administration of ODSO has a significant antihyperglycemic effect in nondiabetic and streptozotocin (STZ)-diabetic rats overloaded with d-glucose. This effect was statically similar to that of glibenclamide. ODSO inhibited the liberated d-glucose intestinal absorption in situ; this inhibitory effect of the intestinal absorption of d-glucose was lower than that of phlorizin (a specific inhibitor of intestinal SGLT2). Moreover, ODSO blocked the intestinal passage of glucose by blocking the transporter SGLT1. While this oil does not show any risk of hypoglycemia on the basal glycemia in healthy rats fasted overnight, glibenclamide, on the other hand, shows a risk of hypoglycemia in nondiabetic rats. Glibenclamide is associated with a higher risk of hypoglycemia in seniors under routine care [47]. The results obtained about the ODSO effect agree with those found by Berraaouan et al. (2014) [34]. They showed that *Opuntia ficus-indica* seed oil has an antihypoglycemic effect, and they explained experimentally that this effect could be related to blocking the passage of d-glucose from the intestine to the bloodstream. Besides, ODSO inhibited the sucrose degradation by intestinal α-glucosidase and the starch degradation by pancreatic α-amylase in vitro and in vivo. In general, the effect of ODSO remains lower than that of acarbose, which is a powerful and specific inhibitor of these digestive enzymes. Therefore, the antihyperglycemic effect might be related to these effects. The acute toxicity test showed that the oil is not toxic, even at 7 mL/kg.

It has been declared that the oil of *Opuntia dillenii* contains a large amount of unsaturated fatty acids, wherein linoleic acid is the major polyunsaturated fatty acid and oleic acid is the main monounsaturated fatty acid. Furthermore, β-sitosterol is the sterol marker, and the unsaponifiable fraction was represented by only γ-tocopherol [48,49]. The oil from *Opuntia* seeds has been found to have an antihyperglycemic effect [34,50]. The phytosterols were found to have an important inhibitory effect on the pancreatic α-amylase and intestinal α-glucosidase activities [50,51,52,53]. Additionally, phytosterols are characterized by the property of increased peripheral use of d-glucose and stimulated insulin secretion [54,55].

Insulin resistance and/or insulin deficiency are the two major defects that cause hyperglycemia in patients with diabetes [56]. The pancreatic β cell increases its insulin secretion when stimulated by d-glucose under the effect of omega-3 fatty acids [57]. Besides, the fluidity of the cell membrane and the regulation of the GLUT4 transporter expression is improved by the polyunsaturated fatty acids [58]. The letters raise the amount of glucose absorbed by 3T3-L1 adipocytes by raising the number of GLUT4 and GLUT1 transporters [59]. β-sitosterol was found to exhibit significant hypoglycemic activity in normal and hyperglycemic models [60].

Another study reported that ODSO is rich in phenolic compounds, and 11 phenolic compounds were identified: catechol, cinnamic acid, phenyl propionic acid, psoralen, syringic acid, sinapaldehyde, 3′-*o*-methylcatechin, (+)-gallocatechin, bisdemethoxycurcumin, 4′-*o*-methyl-(−)-epicatechin 3′-*o*-glucuronide, and viscutin 1 [48]. Another mechanism proposed was the inhibition of key enzymes of carbohydrate metabolism, α-glucosidase, and α-amylase by the phenolic compounds present in ODSO [61,62]. These phenolic compounds, which are characterized by antioxidant activity, are known to have antidiabetic activity by regulating the disturbed oxidative medium under diabetic conditions [63,64]. It has been reported that antioxidants protect the sensitivity of L6 muscle cells from insulin [65]. From these data, it can be concluded that the antihyperglycemia effect of ODSO is related to its richness in natural bioactive compounds that have a very important antidiabetic property while reacting alone or in synergy.

The oil extraction yielded 7%, and this was close to the extractions achieved by other studies. Labuschagne and Hugo [66] reported that the oil content in Cactus *Opuntia dillenii* seeds from South Africa was 5.69%, while Chang [67] reported that the oil content in *Cactus* pear seed oil from China was 6.01%. The two results were close to the obtained results from the present study. However, the oil content from an Italian cultivar was 9.14% [68], and the oil content was 11.05% from another cultivar from Tunisia [69]. Compared to other oil seed crops, *Opuntia dillenii* presented a lower oil content. Indeed, higher amounts were recovered from cotton seeds (15–24%), soybean seeds (17–21%), grape seeds (6–20%), and olives (20–25%) [70].

## 4. Materials and Methods

### 4.1. Chemicals and Reagents

The following drugs and solvents were used in this study: d-(+)-glucose anhydrous, sucrose, starch, and phlorizin hydrate were purchased from Sigma Aldrich (Riedel-de Haen, Germany). Glibenclamide, dimethyl-sulfoxide, acarbose, α-glucosidase, and α-amylase were purchased from Sigma Aldrich ( St. Louis, MO, USA); streptozotocin was purchased from Sigma-Aldrich (Hamburg, Germany); sodium pentobarbital was purchased from Ceva Santé Animale (Libourne, France); and the glucose oxidase-peroxidase (GOD-POD) kit necessary for the measurement of blood sugar was purchased from Biosystems (Barcelona, Spain). All chemicals were of analytical grade.

### 4.2. Opuntia Dillenii Fruit Harvest

The fresh fruit from *Opuntia dillenii* used in this study was collected in February 2018 in the Essaouira city Morocco. After its identification by an expert botanist, Mohammed Fennan, from the Scientific Institute of Mohammed 5 University, Rabat, the specimen was deposited at Mohammed First University, Oujda, Morocco, under voucher code HUMPOM 351.

### 4.3. Powder Preparation and Oil Extraction of Opuntia dillenii Seeds

The fruits of *Opuntia dillenii* were peeled, and then, the seeds were separated from the fruit, cleaned with distilled water, oven-dried at 37 °C for three days, and then crushed until a fine and homogeneous powder was obtained. A quantity of 100 g of powder from the seeds of *Opuntia dillenii* was added to 500 mL of petroleum ether, and then, the mixture was agitated for 24 h at room temperature. After filtration, a rotary evaporator was used to remove the organic solvent at 40 °C. The resulting oil was dried and stored at 4 °C.

### 4.4. Experimental Animals

The present study was conducted by using normal adult Wistar rats and Swiss albinos mice from the local animal husbandry of the faculty of Science, Mohammed First University, Oujda, Morocco. The animals were grouped in polycarbonate cages with soft bedding and ad libitum water and food access in an environmentally controlled room (22–26 °C, with a 12/12-h photoperiod). All rats were cared for in compliance with the internationally accepted Guide for the care and use of laboratory animals, published by the US National Institutes of Health, 2011 [71].

### 4.5. Acute Oral Toxicity in Mice

*Opuntia dillenii* seeds oil acute oral toxicity was evaluated using albinos mice (22–32 g body weight, 1.5–2 months of age). Thirty fasted (14 h) mice were put into five groups (3 males and 3 females in separate cages), then treated orally by different concentrations of ODSO (1, 3, 5, and 7 mL/kg) for the test groups or distilled water (10 mL/kg) for the control group. Signs of toxic effects and/or mortality were observed after 2 h and every 24 h during the 14 days after administration.

### 4.6. Hypoglycemic Test in Nondiabetic Rats

This test was carried out according to the method described by Bellahcen et al. (2012) [72]. Normal rats (190–260 g b.w., 2 to 3 months of age) fasted for 14 h with free access to water were used in this test. Animals were arranged into three groups (3 males and 3 females in separate cages). The ODSO group received a single per os seed oil dose (2 mL/kg b.w.), the glibenclamide group (Glib) received a single per os glibenclamide (an antidiabetic drug that acts directly on pancreatic beta cells to induce insulin secretion [73]) oral dose (2 mg/kg b.w.), and the control group received distilled water at a dose of (10 mL/kg b.w.). Treated rats glycemic index was measured before and 30, 60, 120, 240, and 360 min after product administration. After the test, the animals were subjected to unlimited access to food and water in a controlled room and monitored for two hours after, until blood sugar returned to the normal physiological status.

### 4.7. Induction of Experimental Diabetes

Streptozotocin (STZ; Sigma-Aldrich, Hamburg, Germany) recently prepared in a citrate buffer (0.1-M citrate and 0.1-M phosphate, pH 4.5) was injected intraperitoneally into rats (fasted overnight) by a single dose (60 mg/kg) to induce experimental diabetes (diabetes mellitus [74]). However, the animals were hydrated with glucose water during the days following the injection to avoid hypoglycemic seizures and death. Rats with fasting (14 h) blood d-glucose greater than or equal to 150 mg/dL on the seventh day after injection, indicating polydipsia and polyuria, were regarded as diabetic and incorporated into the study.

### 4.8. Antihyperglycemic Study in Nondiabetic and Diabetic Rats

Both normal and diabetic fasted rats (14 h) were randomly divided into three groups (3 males and 3 females in separate cages). Indeed, healthy and diabetic rat control groups received distilled water at a dose of 10 mL/kg b.w. ODSO groups received the oil at a dose of 0.8-mL/kg b.w. for nondiabetic rats and 1-mL/kg b.w. for diabetic rats (ODSO doses used in nondiabetic or diabetic rats were selected following the preliminary testing [34]). The minimum doses were 0.8 and 1 mL/kg that induced a significant antihyperglycemic effect in normal and diabetic rats, respectively). The glibenclamide groups (Glib) received an antidiabetic drug, glibenclamide, at a dose of 2 mg/kg for healthy and diabetic rats. All the animals were orally loaded with d-glucose (2 g/kg) 30 min after intake of the testing product. Glycemia was measured at 0, 30, 90, 150, and 210 min by the glucose oxidase-peroxidase (GOD-POD) method. Indeed, plasma glucose was measured using a commercial enzyme assay kit. Blood was collected from the caudal artery of rats in heparin hematocrit tubes. These were centrifuged in a hematocrit centrifuge at 3000 rpm for 10 min. In a dosing tube, 1000 µL of enzymatic reagent was added to the 10 µL of plasma (Abs plasma) or 10 µL of glucose (1g/L) (Abs standard), and then, the tube was agitated in the vortex and incubated at 37 °C for 5 min. The absorbance was read at 500 nm by a spectrophotometer. The concentration of glucose in mg/dl was calculated by the following formula:

$$Glycaemia\;\left(mg/dl\right)=\frac{\mathrm{Abs}\;\left(plasma\right)}{\mathrm{Abs}\;\left(satndard\right)}\times 100$$

After the test, the animals were subjected to unlimited access to food and water in a controlled room and monitored for two hours, until their blood sugar returned to a normal physiological state. For diabetic rats, they were monitored by insulin injection.

### 4.9. Single-Pass Intestinal Perfusion in Rats

The effect of ODSO on the intestinal absorption of d-glucose in normal rats was tested using the in situ intestinal single-pass perfusion method [34]. Normal Wistar rats (140–300 g) fasted for 36 h were divided into 4 groups (3 males and 3 females in separate cages). The control group was perfused with the d-glucose perfusion solution (1 g/kg). The Tween 20 group was perfused by the d-glucose perfusion solution (1 g/kg) + Tween 20 (1%). The ODSO group was perfused by the d-glucose perfusion solution (1 g/kg) + ODSO (1 mL/kg). Tween 20 is an agent that was used to solubilize the oil in the perfusion solution, and this group was added to show that this agent does not affect the intestinal absorption of glucose. The phlorizin group was perfused by the d-glucose perfusion solution (1 g/kg) + phlorizin (0.2 mM). Phlorizin is a competitive inhibitor of SGLT1, because it competes with d-glucose to bind to the carrier, and it reduces the intestinal absorption of glucose, lowering the amount of glucose in the blood [75]. At the end of the experiment, an aliquot of 0.5 mL of the perfusate recovered was taken for the d-glucose determination [76], the length of the perfused jejunal fragment was measured, and the rat (state-anesthetized by pentobarbital) was euthanized. The content of the absorbed d-glucose was expressed as the amount of absorbed d-glucose (mg) by the length of the infused jejunum (10 cm) by the duration of infusion (60 min).

### 4.10. Ussing Chamber Assay

For this experiment, normal fasting mice (16 h) were used after cervical dislocation. The method used was described by Eto et al. [77]. The proximal jejunum (5-cm distal from the ligament of Treitz) was dissected and rinsed in cold saline solution. The mesenteric border was carefully stripped off using forceps. The intestine was then opened along the mesenteric border, and four adjacent proximal samples were placed between the two halves of the modified Ussing chambers (exposed area: 0.3 cm^2^). The tissues were bathed with 3 mL of carbogen-gassed Krebs–Ringer bicarbonate (KRB) solution on each side. KRB solution had the following composition (in mM): NaCl, 115.4, KCl, 5, MgCl_2_, 1.2, NaH_2_PO_4_, 0.6, NaHCO_3_, 25, and CaCl_2_, 1.2. In the solution bathing the mucosal side of the tissue, d-glucose was replaced with mannitol. Both solutions were gassed with 95% O_2_ and 5% CO_2_ and kept at a constant temperature of 37 ± 0.5 °C (pH at 7.4). Electrogenic glucose transport was monitored continuously as the short-circuit current (Isc) by using an automated voltage-clamp apparatus linked through MacLab 8 to a MacIntosh computer (Supplementary Materials Figure S1). The ODSO solution was prepared in Ringer’s solution, and 5% taurocholic acid was used to emulsify the oil in the Ringer. The solution was introduced into the mucosal bath in various concentrations (1000, 100, 10, 1, and 0.1 µg/mL) before the addition of luminal d-glucose; a concentration of (1000 µg/mL) was tested to determine whether or not ODSO was affected. The results were expressed as the difference between the Isc peak caused by glucose (measured after 20 min) and the ISC measured only 5 min after adding the oil extract, followed by the addition of 50-µL phlorizin (PHZ; 0.5 mM) in a unique concentration experience. The percentage of inhibition thus referred to the individual values calculated from the paired controls relative to d-glucose (100%). The short-circuit current (Isc) represented the sum of the net ion flux transported across the epithelium [77].

### 4.11. Inhibition Assay of α-Amylase Activity In Vitro

The α-amylase inhibition activity by ODSO was studied according to the procedure described by Daoudi et al. [78], with some modifications. The assay mixtures contained 200 μL of α-amylase enzyme solution (13 IU); 200 μL of phosphate buffer (0.02 M; pH = 6.9); and 200 μL of ODSO (6.67, 5.26, 3.90, 2.56, 1.27, 0.63, and 0.31 mg/mL, solubilized in DMSO (1%)) and acarbose (6.82, 4.54, 2.27, 0.91, 0.45, 0.23, 0.11, and 0.06 mg/mL); acarbose is a specific inhibitor of intestinal alpha-glucosidase and pancreatic alpha-amylase enzymes [79] and DMSO (1%) is an agent that was used to solubilize the oil in the perfusion solution, and this group was added to show that this agent does not affect the intestinal absorption of glucose or distilled water (control). The mixtures were pre-incubated at 37 °C for 10 min. Then, 200 μL of starch (1%) dissolved in phosphate buffer was added to each tube and was incubated for 20 min at 37 °C. To stop the enzymatic reaction, 600 μL of DNSA color reagent (2.5%) was added. Right after, the tubes were incubated for 8 min at 100 °C; then, they were put in an ice-cold water bath for a few minutes. The mixture was diluted by adding 1 mL of distillate water, and the absorbance was measured at 540 nm. The inhibition percentage was calculated using the formula below:

Inhibitory activity percentage = ((DO Control − DO Test)/ DO Control) × 100(1)

where DO Control: Absorption of enzymatic activity without inhibitor; DO Test: Absorption of enzymatic activity in the presence of oil or acarbose.

### 4.12. Inhibition Assay of α-Amylase Activity in Normal and Diabetic Rats

Nondiabetic and diabetic fasted rats were divided into three groups (3 males and 3 females in separate cages) for each model. The control groups received distilled water at a dose of 10 mL/kg, the ODSO groups received oil at a dose of 0.8 mL/kg, and the acarbose groups received acarbose at a dose of 10 mg/kg by oral gavage. All the animals were orally loaded with starch (2 g/kg) at 30 min after treatments. Their blood was taken by the venous section of the tail from rats under light ether anesthesia at 0, 30, 60, and 120 min to measure glycemia. After the test, the animals were subjected to unlimited access to food and water in a controlled room and monitored for two hours, until their blood sugar returned to a normal physiological state. For diabetic rats, they were monitored by insulin injection.

### 4.13. Inhibition Assay of Intestinal α-Glucosidase Activity In Vitro

The effect of the ODSO against intestinal α-glucosidase activity was quantified colorimetrically by monitoring the d-glucose release from sucrose degradation, according to the protocol described by Ouassou et al. [80], with some modifications. The assay mixtures contained 100 μL of sucrose (50 Mm), 1000 μL of phosphate buffer (50 mM; pH = 7.5), and 100 μL of intestinal α-glucosidase enzyme solution (10 IU). Ten microliters of distilled water (control, acarbose (Acarb), and ODSO solutions at different concentrations (40.75, 81.5, 163, 326, and 652 µg/mL solubilized in DMSO (1%)) or DMSO (1%) were added to the mixture. Then, it was incubated at 37 °C in a water bath for 25 min. Then, the mixture was heated at 100 °C for 5 min to stop the enzymatic reaction, and the released d-glucose was estimated by the d-glucose oxidase method using a commercial auto-kit. The absorbance was measured at 500 nm, and the inhibition percentage was calculated using the formula below:

Inhibitory activity percentage = ((DO control − DO Test)/DO control) × 100(2)

where DO Control: Absorption of enzymatic activity without inhibitor; DO Test: Absorption of enzymatic activity in the presence of oil or acarbose.

### 4.14. Kinetics of Intestinal α-Glucosidase Inhibition In Vitro

In this study, the type of inhibition of ODSO against intestinal α-glucosidase was determined. Indeed, a constant concentration of intestinal α-glucosidase was incubated with increasing amounts of its sucrose substrate (2, 4, 6, 8, and 10 mL/L) in the presence or absence of two concentrations of ODSO (solubilized in DMSO (1%)) (165 and 365 μg/mL). The optimal dose used in this study was determined by testing the activity of this enzyme as described earlier and from a preliminary test of the kinetics of the intestinal α-glucosidase inhibition. Moreover, the mode of inhibition of this oil was determined by analyzing the Lineweaver-Burk representation of the results, which was calculated from the results according to Michaelis-Menten kinetics [81].

### 4.15. Inhibition Assay of Intestinal α-Glucosidase Activity in Normal and Diabetic Rats

Nondiabetic and diabetic fasted rats were regrouped into three groups (3 males and 3 females in separate cages) for each model. The control groups received distilled water at a dose of 10 mL/Kg, the ODSO groups received oil at the dose of 0.8 mL/kg, and the Acarb groups received acarbose at the dose of 10 mg/kg by oral gavage. All the animals were orally loaded with sucrose (2 g/kg) 30 min after treatments. Their blood was taken by the venous section of the tail from rats under light ether anesthesia at 0, 30, 60, and 120 min to measure their glycemia. After the test, the animals were subjected to unlimited access to food and water in a controlled room and monitored for two hours, until their blood sugar returned to a normal physiological state. For diabetic rats, they were monitored by insulin injection.

### 4.16. Statistical Analysis

Data were presented as the mean ± standard errors and were subjected to statistical analysis using GraphPad Prism 5 (GraphPad Software), San Diego, CA, USA. Multiple-group comparisons were analyzed by one-way analysis of variance (ANOVA). Statistical significance was accepted as *p* < 0.05.

## 5. Conclusions

This study first explored the mechanism of action of the antidiabetic effect of the ODSO. This oil exerted an antihyperglycemic effect in healthy and STZ-provoked diabetic rats. This effect was due, in part, to the inhibition of the intestinal α-glucosidase and pancreatic α-amylase enzymes, as well as the inhibition of intestinal d-glucose absorption. Further studies are needed to explore other pathways through which this antihyperglycemic effect passes.

## Figures and Tables

**Table 1:** Kinetics parameters of α-glucosidase inhibition by *Opuntia dillenii* seed oil (ODSO).

ODSO Concentration (μg/mL)	Km (mM)	Vmax (μM/min)
0	71.42	166.66
165	71.42	166.66
325	71.42	83.3

## Data Availability

The data presented in this study are available on request from the corresponding author.

## Supplementary Materials

The following are available online, Figure S1: Ussing chamber assay.

## Sample Availability

Sample are available from the authors upon reasonable request.
