# Supplementary figures and images for: Opuntia dillenii (Ker Gawl.) Haw., Seeds Oil Antidiabetic Potential Using In Vivo, In Vitro, In Situ, and Ex Vivo Approaches to Reveal Its Underlying Mechanism of Action
Source: Molecules. 2021 Mar 17;26(6):1677. doi: 10.3390/molecules26061677 (PMC8002680; doi:10.3390/molecules26061677)

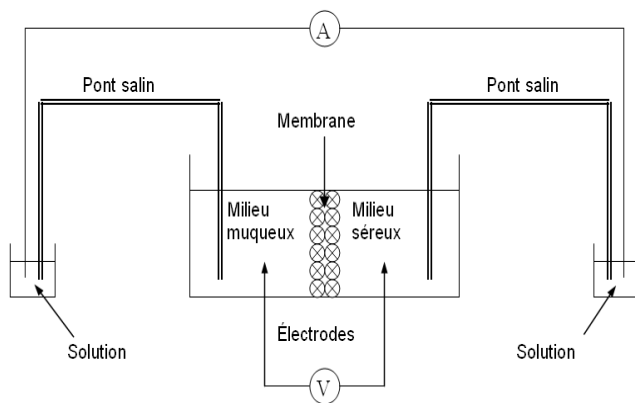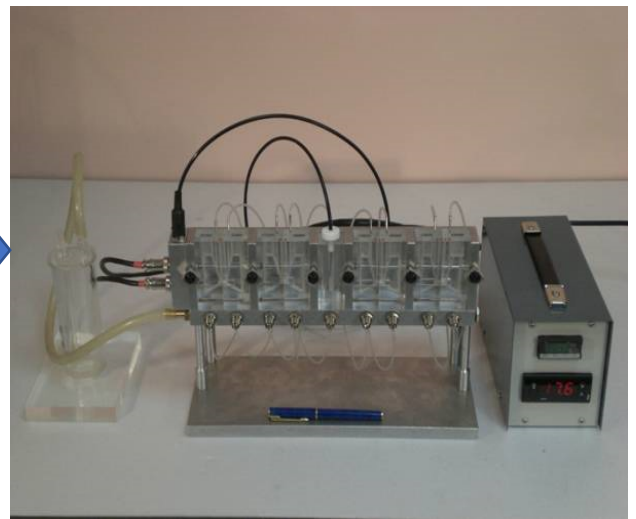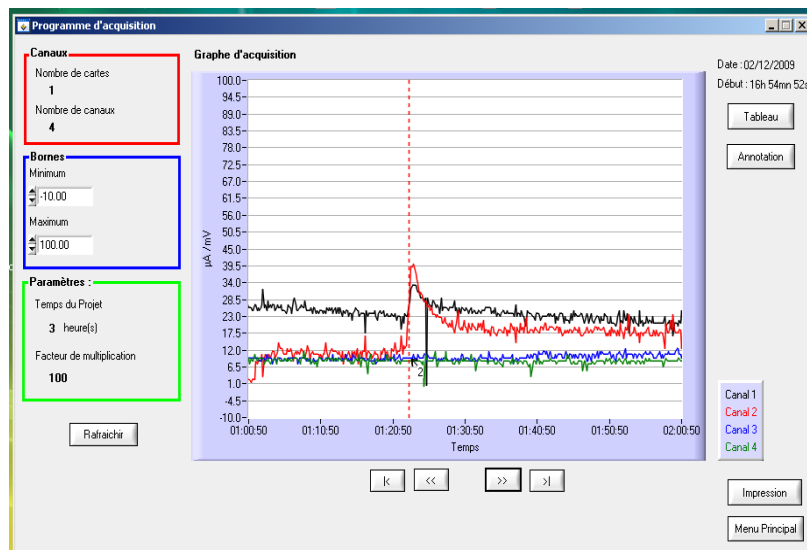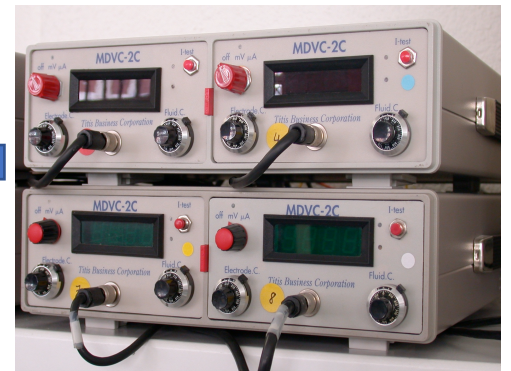

## Ussing Chamber assay

Supplement: Supplementary file 1 [file molecules-26-01677-s001.pdf]
